# Spinal Arthropathies

**Published:** 1875-05

**Authors:** S. Weir Mitchell

**Affiliations:** Member of the National Academy of Sciences


### SPINAL ARTHROPATHIES.

By S. WEIR MITCHELL, M.D.,

Member of the National Academy of Sciences.

It has been my good fortune within the last two years to meet with a number of cases of disease of the joints of the legs, associated in each instance with atrophic states of limited groups of muscles, and offering more or less distinct clinical evidence of being due to spinal disease. I shall have occasion, as I proceed, to show how these cases differ from any as yet described, both in their early symptoms, and in their more favorable prognosis, but I shall perhaps make clearer my meaning, if first I call attention, however briefly, to the history of spinal and neural arthropathies, a subject which owes its best study to American and French students. The history of spinal arthropathies is well told by Charcot, in his *Lecons sur les Maladies du Systeme Nerveux*, Paris, 1872–73, p. 100 et seq. This author distinguishes joint lesions of spinal origin as of two varieties. Those which are acute or subacute and are accompanied with redness, swelling, and sometimes with more or less violent pain. The second class he describes as chronic, of slow growth, and, as I shall point out, as being remarkably different from the first-named disorders.

The history of this subject is somewhat interesting, and the more so, because to an American physician belongs the long-forgotten credit of the first discovery that "an obvious spinal cause may produce a rheumatism characterized by heat, pain, redness, and tumefaction." The quotation is taken from the second paper on rheumatism by my father, the late Dr. John K. Mitchell. In his first essay he described cases of Pott's disease, in which, below the diseased region, there were acute inflammations of the joints, which proved amenable to treatment directed to the point of spinal lesion.* M. Charcot, *op. cit.*, pp. 100, 101, in acknowledging the first mention of these facts, adds, correctly, that this cause of arthropathies is rare, and, apparently ignorant of Dr. Mitchell's second paper,† says that traumatic lesions of the cord are more often the parent of joint disorders, and refers to Gull‡ for instances of spinal commotion as competent to occasion a like result. Until in my work on injuries to nerves I recalled attention to the true author of this clinical discovery, Gull has usually had credit for being the first original observer of these interesting facts, while actually the credit is due to the American author. In his second paper Dr. Mitchell describes the very curious case of Dr. Parker, of Elkton, Maryland, in October, 1831. This gentleman, who had previously had rheu-

* Am. Journ. Med. Sci., 1831, p. 55.
† Am. Journ. Med. Sci., 1833, p. 360.
‡ Guy's Hosp. Repts., 3d series, t. iv.

matism, was thrown from his carriage, and, falling on his back, was instantly paralyzed slightly in his arms, but totally in his lower limbs. The next day he had swelling, pain, redness, and heat in the joints of the hands and wrists. These symptoms were thereafter aggravated by pressure upon the seat of injury in the spine, but relieved by the application of certain remedies to the spine. They shifted their place from joint to joint in the upper limbs, but did not affect the legs.

In 1846, Dr. Scott Alison * described very clearly the arthropathies which occur in hemiplegia, but incorrectly ascribed them to the lithic diathesis under which he presumed his patients to be suffering, and to which he conceived all such cases must be due. Since then, Brown-Sequard, Charcot, and the author, as well as some others, have recalled attention to hemiplegic arthropathies. Like forms of joint disease have been seen in myelitis, and as results of spinal tumors, while in locomotor ataxia, Charcot has delineated with great fidelity a more chronic but not less troublesome form of joint disease.

According to Charcot, similar lesions have been seen in progressive muscular atrophy by Patruban, Remak, and Rosenthal, but I have been unable to procure the papers referred to. Finally, in 1864, I described, in conjunction with Drs. Morehouse and Keen, the joint diseases caused by injuries to nerve trunks, and again and again since I have illustrated anew this clinical sequence, by numerous reports of cases of manifold forms of nerve injury.

The pathological mechanism of the various neural arthropathies is clear only up to a certain point, beyond which all is as yet obscure. It has been made most probable, that all the spinal arthropathies are due to disease of the gray matter of the anterior cornua of the cord. It has been shown that this disease of the gray matter is not caused by the inertia to which some forms of spinal disease condemn the patient, and that neither in these cases are the peripheral nerves at fault. It has also been suspected that the cerebral arthropathies are caused by a descending sclerosis finally involving the anterior tracts of gray matter in the cord, but as to this I have a reasonable doubt, owing to the very early date at which I have sometimes seen joint lesions follow a cerebral hæmorrhage,

* Lancet, 1846, vol. 1, p. 227.

and to the suddenness of production, and the great generalization of the joint lesion in some of the same class of cases. It has been remarked that the joints swell from fifteen days to three months after an apoplectic attack, coincidently with the coming on of the "late rigidity," which we now commonly ascribe to descending sclerosis. I have, however, again and again seen the joint lesions come on earlier, and without any muscular rigidity, and I have also seen them get entirely and rapidly well, which does not look as if they could in these cases have been due to sclerosis. Thus in one case, they came on the day after an attack of left hemiplegia, in another on the third day.

The local peculiarities of most of the spinal and cerebral arthropathies are not such as enable the most acute clinical observer to distinguish them from some of the ordinary types of rheumatism, a fact which certain authors have frankly admitted. The general clinical characters, says Charcot, which differentiate them from common rheumatism of joints, are their limitation to the joints of the members afflicted with palsy, their relation in time to hemiplegia, the coexistence of other trophic troubles ; but, if we begin by suspecting that some at least of our general rheumatisms may be of spinal birth, these would be only points in our favor, and the cases of Pott's disease in which remote joint lesions follow it, without palsies, would enable us to believe that such a spinal condition from disease might sometimes exist as would give rise to rheumatic joints without the concurrent existence of other troubles more palpably of neural origin. Moreover, it has occurred to me twice, to see cases of chronic rheumatism following acute rheumatism where an apoplectic attack produced a few days later an enormous exaggeration of the joint disease on the palsied side, so that there is something in the status of a palsied limb which favors the increase of an already present rheumatism.

Yet, however he may differ as to whether rheumatic fever be ever a spinal disease, all pathologists now admit the existence of joint disease distinctly due to neural lesions, but as to the mechanism of the production of these arthropathies, we are still at fault. Our discovery of arthropathies caused by injury of nerve trunks, seemed to promise to make the research more simple, but as yet it has done little to aid us. It has been made clear, however, that in these, as probably in central lesions, they are due rather to irritative states than to absolute defects of power. It has been shown that they are caused neither by vascular palsies nor by vasal spasm, nor yet by inertia, which is, as we know, competent to cause, in limbs long at rest on splints, certain chronic forms of joint disease. I have certainly seen neuro-traumatic joint disease break out suddenly and with terrible severity within three days of a nerve lesion, which but slightly affecied either motion or feeling, and which certainly gave rise to no atrophies, so that it also seems unfair to attribute them to defects of nutrition in this sense or in this direction.

I have elsewhere pointed out how curiously even very slight lesions of nerves affect the cutaneous secretions, and it seems fair to infer that disturbances in the chemical balances of the deeper tissues may likewise arise from as slight neural causes. How far these may also come from central disease, and how competent in either case they may be to trouble the life of the tissues and occasion local inflammations, we can hardly yet determine. It is but an hypothesis, yet of some value as giving a fresh direction to research. It is indeed hardly possible to refrain from speculation upon a subject at once so open and so interesting.

I have seen but one case of entire annihilation of nerve influence in a limb, and in this all the nerves were cut save the fibres which pass with the vessels. No notable joint lesions followed the section. The sections of single nerves never altogether insulate neurally a part even of a limb or lesser member,* but it is commonly the partial sections of one nerve which cause joint disease, and then arises this curious question: Do they act directly along the injured peripherally distributed fibres and thus affect the joint, or does the local irritation influence the centre, and through it and the still entire nerve threads act upon the joint to disorder its nutritive life? I incline to this latter opinion, which is favored by various reasons, and especially by some of the cases I have elsewhere reported. But if this view be taken and we come to conceive of a state of the centres in which there were disturbance from without competent to put them in a state to cause joint lesions, we are readily made able to add

* Section of a nerve always leaves the joint in relation with other undestroyed branches.

another step in belief, and conceive that sometimes these centres may without peripheral irritations be thrown into such a state as to occasion these lesions.

Hitherto, in all of the reported cases of neural arthropathy, there has been a passive central or peripheral nerve lesion, and usually there have been also precedent symptoms not related to the joints, such as atrophy, paralysis, anæsthesia, or hyperæsthesia. In three of the four histories which I shall here relate, the joint lesion came first, existed alone for a time, and was followed by other nutritive, sensory and motor conditions of the limbs, which revealed the spinal column as the organ upon which the whole chain of phenomena depended. Surely this is a fact of great pathological significance, since it is open to suspect that if the spinal lesion had been checked at a certain point we might have had only the joint disease, or such slight derangements in the way of numbness or lack of power as might readily escape notice, or as often are seen by acute observers in rheumatisms suspected by no one to be of neural birth. One possible fallacy may exist to mar this view of the pathogenesis of some arthropathies, and to it I shall by and by refer.

CASE I.—Mr. B., of E. Pennsylvania, consulted me last year in the autumn, on account of a painful affection of the right knee-joint. Mr. B. was a bank officer, æt. 48, of slight figure, free from previous disease, and of untainted descent. In the spring of 1873 he was subject to severe mental and moral strain, and for some weeks to unusual exertion a-foot. In June he had slight pain in the dorso-lumbar spine, which was eased by rest. The accompanying sense of lassitude left him after a short summer holiday, but early in September he began to have pain and swelling and stiffness in the right knee, and at length was forced to remain at rest, the knee having been put in a splint. Despite pretty active treatment it grew worse, and he was led to consult me in November.

At this time the leg presented nothing abnormal save in the joint. There was no wasting, no loss of feeling or of electrical reactions. I advised absolute rest, pressure by sponges and bandage, tonics, and moderate doses of iodide of potassium. The joint was hot and largely swollen, the patella lifted by effusion, and the pain severe especially at night. He came back to the city within a month. At this time there was pain and slight tenderness on the right of the eighth and down to the twelfth dorsal vertebra inclusive, an aching sense of distress over which ice caused a feeling of burning. The joint was in all respects better, but still swollen slightly, red, and painful and tender. The thigh and leg presented a curious change. The extensor group in the thigh was wasted at least one-half, the peroneal and gastrocnemial groups were similarly altered, but to a less degree. All of these muscles were much enfeebled, and responded only to galvanic currents of at least thirty cells, and best to ascending currents. The sensibility of the skin of the leg below the knee was much impaired, that above the knee but slightly lessened.

I was amazed to find so remarkable a change in so short a time, and was fortunate in having made previously so complete an examination as to feel sure that when first I saw the case the joint lesion stood alone.

For a few days I enjoined rest, with cut cups thrice to the spine. The local relief was great, and although no other treatment was then employed, a change for the better was seen at once in the joint. After a time I began to treat the knee with powerful galvanic currents, and the muscles with reversed galvanic currents. The most rapid improvement followed, and after thirty sittings I found that I got ready responses by induced currents which were thenceforward used every day. The splints were early laid aside, slight movement permitted, and when only some feebleness of gait remained, I employed hypodermic injections of strychnia.

After three months Mr. B. went home well. He has since had a short relapse, with a display of all the same symptoms in a lesser degree, but the same treatment readily overcame them, and a course of cod-liver oil and iron, with some changes in his ways of work, has sufficed to preserve him in health up to this date.

Case II.—My second case was a woman, æt. 32, from Wilmington. She was at the head of a busy millinery business, and had been in good health, and free from pain. In the spring of 1873 she began to have pain in the left knee-joint, and after suffering some months, gradually grew more and more feeble as to the use of the left leg. In the autumn she applied to me, and was then in a pitiful state of pain and lameness. The joint was enormously swollen, and very painful, as well as most curiously tender. The temperature was two degrees above that of the right knee, and the patella could be rocked on the distended joint. To my surprise, the whole anterior group of extensors of the leg was wasted at least one-half, and could not be stirred by the will or by any form of current, galvanic or induced, nor yet through the nerve trunks.

The sensibility was, however, unimpaired. The back was free of tender spots, the general health fair, but not vigorous, and there was no functional disturbance of stomach, kidney, or generative organs.

I treated this case as I had done the other, by galvanism until induction currents acted, and thenceforward by these latter. The general treatment consisted in the use of tonics and full doses of strychnia. The gain was sudden and steady, so that I was able, after a few weeks, to leave the case in the hands of my friend, Dr. John K. Kane, of Wilmington, under whose charge she continued to improve, so as within a few months to be entirely well.

It is, of course, possible that both of these cases may have been joint troubles originating without neural cause, and producing by reflected irritations muscular losses; or more directly giving rise to an ascending neuritis competent in time to occasion like results. The nerve tracks, however, were searched again and again in both cases for tenderness, and always in vain, while the spinal symptoms of the first case seem to have been distinct, so that, on the whole, I reached the conclusion that in both there was a limited spinal lesion, and if so in both (and here is their peculiarity), the joint disorder came first, and for a time stood alone.* The next case, as to the spinal birth of which no shadow of doubt can exist, enables us to feel far more sure that the two cases first given had also this origin. It has also this added value, that it was seen and studied by others besides myself, none of whom had finally any other view.

Case III.—The subject of the following most remarkable history is the wife of a physician of distinction in

* The very remarkable wasting of muscles in some joint diseases, as of the hip, seems to be due to reflected irritations and not to mere inertia, but these wasted muscles usually react under electric currents as well as their healthy fellows.

a neighboring city—a woman of unusual energy and intelligence, and previously in good health.

On April 18, 1870, she first felt a slight sense of lameness in the left knee. It caused annoyance in going up or down stairs, in sitting or kneeling, far more than in walking. On the 23rd an examination revealed the presence of too much fluid in the bursa, and on the 24th the joint was distinctly swollen, and there were slight pains at times down the inside of the leg. The knee had gained in one day three-fourths of an inch in girth. So great was the tension and pain, that on the 25th absolute rest was ordered, and leeches were applied. The bleeding continued too long, and seemed to cause unusual feebleness, but on the 26th the inflammation was much lessened. Stimulants were used internally, as she seemed singularly weak, and wet cold was applied locally. A day or two later dry cold was used, and the knee was found to be easy if at rest, but very painful when stirred or even handled.

On May 9th for the first time in trying to move the leg, which by this was much more free of pain, Mrs. B. noticed a want of power to lift the limb, or to change its place from side to side. The extensors, abductors and adductors were enfeebled.

At this time the leg was put on a gutta percha splint, and kept on it two weeks with compression to the knee by sponges and bandages, while locally iodine was used.

On June 1st Dr. J. H. Brinton asked me to visit Mrs. B. in consultation. At this time there was some pain in the lumbar spine—not a very definitely fixed pain—and there were slight twitches in the thigh muscles above mentioned, and also in the peroneal group—this symptom being worse at night and very disturbing to the patient—as they sometimes moved the patella so as to give pain.

On April 24th the knee measured 14½ inches; and during June it continued to be 13 inches. The other measurements will, by and by, be mentioned; they showed at this time remarkable atrophy of the whole limb, but chiefly of the anterior groups of muscles, and there was entire loss of power to lift the leg or even to stir the extensors, or to move the part laterally; yet the joint was vastly better, and indeed quite free from pain, a slight roughness being present when it was bent. The gain took place under use of cod-liver oil, iron, shampooing, and induction currents, which at this time failed to stir the disordered muscles. The temperature of the limb had fallen ever since the loss of power began, but, with the improvement alluded to, the limb throughout became warmer.

*July* 1. The foot could be rested on the floor with the knee in half flexion, and July 15th could be voluntarily raised from the floor a little, while extended.

*17th.* Mrs. B. was carried out of town, and finally spent the summer at the sea-side, continuing her treat ment with daily sea-baths.

During July she walked on crutches, and on August 25th was able without crutches to walk a few steps, though not without limping and pain in the knee. Meanwhile the gain as to temperature, power and nutrition continued, and the sensibility, never wholly lost, became decisively better.

*Sept.* 5. I saw her on her return. She could now walk about one-fourth of a mile on crutches.

The measurements were as follows:—

| | Over patella. | 3 inches above patella. | 8 inches above patella. | 4 inches below patella. | 8 inches below patella. |
|---|---|---|---|---|---|
| June 3, 1870. | 13 | 13 | 16 | 10¼ | 10½ |
| Sept. 16, 1870. | 13⅞ | 14½ | 17½ | 11½ | 11 |

June 3, 1870. Ankle, 6¾. Sept. 16, 1870. Ankle, 6¾.

During the autumn the improvement went on, and in November, Mrs. B. could walk a few yards without aid, but both in the winter and through the last summer it was curious to see how all of the symptoms fluctuated almost from day to day.

Early in the winter the other knee began to suffer, and precisely the same set of symptoms were seen in the right knee and leg, save that the loss of sensation was not so considerable. I had now, of course, no longer any remainder of doubt as to the spinal origin of this most interesting case.

With varying fortunes Mrs. B. passed through the winter, the joints becoming worse at times and again better, but every new onset of arthritic trouble being followed or accompanied by increase of atrophy, loss of power and sensation, and the limbs being liable to notable alterations in temperature.

*June* 1, 1871. Mrs. B. saw Dr. Brown-Sequard, to whom I wrote an account of her case. Under his advice she took iodide of ammonia and strychnia in increasing doses, and with these aids and steady sea-bathing became vastly better, and in the fall could walk a mile without aid. The limbs now showed a new increase in size and firmness, sensation was almost perfect, and the joints free of pain. The winter brought, as before, some return of trouble, but not to the same extent as in the last winter.

*April* 1, 1872. Dr. Brown-Sequard met me in consultation, when, except the use daily of ice rubbing for the knees, no change was made.

The summer of 1872 was spent at Cape May, with the usual good effects. In October, Mrs. B. was able to walk about in-doors and out much as other women, all of her untoward symptoms having disappeared. In 1873 she went to Pittsburg to reside, and while there had no further annoyance until January, 1873, when she had a most curious and instructive attack of the old symptoms, accompanied with general feebleness, and the appearance of an eruption of herpes, which, originating on the left shoulder under the left arm, passed over the left chest. The eruption was very painful, and lasted about ten days. I did not see her in this attack, but Dr. Benham, her physician, has been so kind as to give me a full description of this incident of the case. Previous to the attack there had been some causes of weakness. The palsy and knee troubles passed away under use of oil and strychnia, and now, except as to power to kneel or stoop readily, Mrs. B. is perfectly well.

In this remarkable history of joint lesions, atrophic palsy preceded by twitching of the muscles, dysæsthesia, and altered thermal conditions, we have all the needed evidence to show that there was a central cause, and that this was a local myelitis of the spinal cord. Its clinical value lies in the fact that the joint disease distinctly preceded the remaining symptoms.

I have met with other examples of spinal disease producing arthropathies, but in all of them the spinal malady existed for long periods before it gave rise to arthritis. These histories, therefore, will teach us nothing new, and I pass on to the last, and perhaps the most remarkable of my cases. In November of last year, Dr. Bolling, of Chesnut Hill, asked me to see with him a case which had been looked upon as mere rheumatism, but which Dr. Bolling had rightly concluded to be of neural origin.

CASE IV. — The patient, Eliza M., æt. eight years. When six years of age, in May, began to complain of pain in both ankles, and to turn the feet under her in walking; braces were applied, with no relief to the pain, and they were at length removed. The trouble, nevertheless, grew better, to reappear the following spring, with pain both in the ankles and wrists. Then suddenly the toes swelled at their joints, becoming red, tender, hot, and stiff. Within a few days the ankle-joint also became intensely inflamed, and finally the whole of both feet. Neither the knees nor the hands became inflamed. As the diseased joints grew better, which they did very rapidly during six weeks at the sea-shore, the feet were found to drag in walking, although after a time she became quite well and even active. During each of these attacks, the peroneal and anterior tibial muscles and the interossei of the feet became singularly wasted, and after each onset all of these muscles underwent within a few weeks the most amazingly rapid repair. The hands passed through like changes, but their joints became stiff, and a little rough and enlarged, without any pain, and the interossei, the thenar group, and the extensors exhibited the same speedy wasting, and the same as sudden and remarkable restoration, but in all the attacks this revival left them not quite well nor fully active, so that after each return of disease there was a slight addition to the permanent disability.

As to the latest attack, in November, 1874, I got a better account. The child when seen by me was healthy looking, cheerful, and bright. About November 10th, she began to drag the feet unusually. Then a few days later, she had nausea, and occasionally sick stomach, which endured for a week, whilst meanwhile pain appeared in the leg and in both feet, together with intense pain, heat, redness, and great swelling of all the joints of both feet. The pain was very great, and the tenderness on motion distressing; Dr. Bolling thought that taken alone, no better illustration of acute rheumatism could have been found.

The joints of the hands underwent their usual changes, becoming deformed without pain. When I first saw her in November, the appearances described were lessening in severity, but the atrophies were well marked.

Dec. 10, 1874, Dr. Bolling met the patient at my house, when I made with his aid the following careful notes of her condition. The hands present good types of the "claw hand." The first phalanges are drawn back in extreme extension, the second and third phalanges flexed. The interossei wasted the first most notably. The thumb nails look directly upwards, so as to be on the same plane as those of the fingers, owing to wasting of the whole thenar group of muscles. The palm is flattened. All of the joints are large and stiff. These peculiarities Dr. Bolling tells me nearly altogether disappear between the attacks. The feet are cold, 85° F., bluish and congested. The joints have already lost almost all traces of disease, and are only a little stiff and swollen. The intrinsic muscles of the feet and the flexors of the feet are wasted, but the extensors of the feet, normal as to size, are slightly contracted in tonic spasm. The toe nails are curiously and deeply indented by numerous transverse furrows, probably marking interruptions of growth.

All the foot extensors are palsied, but there is still feeble power to flex and extend the toes, and extend the feet. The interossei of the hand and the extensors of the wrist are very weak, the common extensor of the fingers weak, the flexors all healthy.

Sensation as to touch, pain, localization, and temperature everywhere normal.

The electrical conditions were interesting. A galvanic current (interrupted and reversed) of 60 cells did not move the flexors of the feet, nor was it capable of moving the gastrocnemius which yet responded to volition. Powerful induced currents also provoked in these parts no reaction. Induced currents moved the third, fourth and fifth interossei of the hands, and best on the right side, but did not stir in either hand the first, second and third interossei, or the thenar group, save only the ulnar adductor, and this feebly. The extensor group in the arms reacted badly under induction currents.

There seemed to be no other muscular loss or defect, and the heart, kidneys, stomach and special senses were normal.

The symptomatology of this case of course allies it with progressive muscular atrophy, from which, however, it is set apart by most obvious clinical peculiarities.

It is said that an elder sister died of the same disease, and I ought to add that both parents are unusually healthy people.

The last case of my series is in many points different from the others, but more especially in the oddity of the hand symptoms. Apart from this portion of the case, it resembles, save in the acuteness of attack, the three others; but in it the loss of power preceded the arthritic symptoms. In the others there was joint disease of painful character, followed by atrophies, loss of power, and change of temperature, while in one only was there also loss of feeling. In all, the changes were rapid, and the cure of the wasting and palsy speedy and complete.

